# The development of a C5.0 machine learning model in a limited data set to predict early mortality in patients with ARDS undergoing an initial session of prone positioning

**DOI:** 10.1186/s40635-024-00682-z

**Published:** 2024-11-14

**Authors:** David M. Hannon, Jaffar David Abbas Syed, Bairbre McNicholas, Michael Madden, John G. Laffey

**Affiliations:** 1 Department of Anaesthesia, Galway University Hospital, and School of Medicine, University of Galway, Galway, Ireland; 2 Anaesthesia and Intensive Care Medicine, School of Medicine, University of Galway, Galway, Ireland; 3 School of Computer Science, University of Galway, Galway, Ireland

**Keywords:** ARDS, Prone positioning, Machine learning, Critical care, Mechanical ventilation

## Abstract

**Background:**

Acute Respiratory Distress Syndrome (ARDS) has a high morbidity and mortality. One therapy that can decrease mortality is ventilation in the prone position (PP). Patients undergoing PP are amongst the sickest, and there is a need for early identification of patients at particularly high risk of death. These patients may benefit from an in-depth review of treatment or consideration of rescue therapies. We report the development of a machine learning model trained to predict early mortality in patients undergoing prone positioning as part of the management of their ARDS.

**Methods:**

Prospectively collected clinical data were analysed retrospectively from a single tertiary ICU. The records of patients who underwent an initial session of prone positioning whilst receiving invasive mechanical ventilation were identified (*n* = 131). The decision to perform prone positioning was based on the criteria in the PROSEVA study. A C5.0 classifier algorithm with adaptive boosting was trained on data gathered before, during, and after initial proning. Data was split between training (85% of data) and testing (15% of data). Hyperparameter tuning was achieved through a grid-search using a maximal entropy configuration. Predictions for 7-day mortality after initial proning session were made on the training and testing data.

**Results:**

The model demonstrated good performance in predicting 7-day mortality (AUROC: 0.89 training, 0.78 testing). Seven variables were used for prediction. Sensitivity was 0.80 and specificity was 0.67 on the testing data set. Patients predicted to survive had 13.3% mortality, while those predicted to die had 66.67% mortality. Among patients in whom the model predicted patient would survive to day 7 based on their response, mortality at day 7 was 13.3%. Conversely, if the model predicted the patient would not survive to day 7, mortality was 66.67%.

**Conclusions:**

This proof-of-concept study shows that with a limited data set, a C5.0 classifier can predict 7-day mortality from a number of variables, including the response to initial proning, and identify a cohort at significantly higher risk of death. This can help identify patients failing conventional therapies who may benefit from a thorough review of their management, including consideration of rescue treatments, such as extracorporeal membrane oxygenation.

This study shows the potential of a machine learning model to identify ARDS patients at high risk of early mortality following PP. This information can guide clinicians in tailoring treatment strategies and considering rescue therapies. Further validation in larger cohorts is needed.

**Supplementary Information:**

The online version contains supplementary material available at 10.1186/s40635-024-00682-z.

## Introduction

Acute Respiratory Distress Syndrome (ARDS) refers to a constellation of changes relating to widespread inflammation of the lungs. It leads to severe, often life-threatening, problems with the normal functions of breathing [1]. ARDS is common, complicating approximately 10% of all Intensive Care Unit (ICU) admissions, and almost 25% of patients who require mechanical ventilation [2]. It has a high associated mortality of 35–65% [3].

The key pathophysiological implication for lungs that are injured with ARDS is an inability to oxygenate the blood effectively. Clinicians often use the ratio of oxygen partial pressure in arterial blood to inspiratory fraction of oxygen (the P/F ratio) as a surrogate for pulmonary shunt fraction [4], and to classify the severity of lung injury [5]. The gold-standard measurement of this parameter requires the insertion of a pulmonary artery catheter (PAC), but this is associated with significant complications [6, 7].

The mainstay of therapy for ARDS is to treat the underlying cause that has driven the process within the lungs and provide supportive therapy to the patient. The key respiratory therapy in ARDS consists of mechanical ventilation (MV) [8]. Placing a patient in the prone position is another non-pharmacological therapy for ARDS [9]. The prone position leads to changes in the compliance of the lung and chest wall [10], resulting in more homogenous distribution of stress and strain forces in the lung [11]. This results in more homogenous inflation of lung tissue [12] and better ventilation–perfusion matching [13]. The result is improved oxygenation of the injured ARDS lung [14]. Evidence from clinical trials suggests that the prone position can reduce mortality when performed for moderate–severe ARDS, early in the course of the disease (≤ 2 days), for at least 16 h [15].

Identification of patients with ARDS at high risk of early mortality would enable early thorough review of all aspects of management including consideration of alternative treatments such as extra corporeal membrane oxygenation (ECMO) that might otherwise be delayed. It is likely that early initiation of ECMO is most beneficial [16]. One modality that could assist in predicting early mortality risk is machine learning (ML), which has shown promise in predicting the development of ARDS [17], and the development of new indices to diagnose ARDS [18]. These techniques have also been used to identify phenotypes within ARDS [19], and clarify how certain phenotypes of COVID-19 ARDS can respond to steroid treatment [20]. To our knowledge, no model has been developed to predict early mortality in the context of patients with respiratory failure managed with a regimen that included prone positioning.

### Research aim

This study aimed to develop a prognostic model to predict early (7-day) mortality following an initial management regimen including prone positioning in invasively ventilated patients with ARDS. This was performed using demographic and physiological data, alongside routine blood tests. The work is reported using the recommendations outlined in the Transparent Reporting of a multivariable prediction model for Individual Prognosis or Diagnosis (TRIPOD) Initiative [21].

## Methods

### Inclusion criteria

The setting for this study was a single centre tertiary ICU at Galway University Hospital in Ireland. Patients who met the following criteria were included:

- patients > 18 years of age.
- met the definition of ARDS as per the Berlin criteria [5].
- ARDS was a result of any aetiology (inc. COVID-19).
- were invasively ventilated.
- underwent at least one session of prone positioning whilst mechanically ventilated.

ARDS was classified as “C-ARDS” if lung injury was secondary to COVID-19, and “ARDS” if it was due to other causes. Severity was stratified as per Berlin criteria [5].

### Clinical aspects

ARDS severity was classified as per the P/F ratio thresholds specified in the Berlin Criteria [5]. Patients were managed as per the latest American Thoracic Society/European Society of Intensive Care Medicine/Society of Critical Care Medicine guidelines [22]. The initiation and cessation of prone positioning was conducted as per the evidence presented in the PROSEVA study [23]. PP was stopped when then patient was deemed ready to commence weaning from the ventilator, when repeated proning was deemed ineffective by the treating clinician, or if the patient became too unstable from a cardiovascular point of view to undergo PP.

### Data

Metavision (iMDsoft, Tel Aviv, Israel), the Clinical Information System (CIS) used in the ICU of the hospital, was interrogated on three occasions on 10/05/2022, 5/9/2022, and 24/11/2022. Data was isolated using an inbuilt Query Wizard and then processed into a time-series format to allow for further processing [24].

Several categories of data were collected. Demographic details were recorded on admission to the unit. Prior to initial proning, the results of blood tests were recorded. This included full blood count (FBC), blood clotting assays, urea and electrolytes (U&Es), and liver function tests (LFTs). Ventilator settings were noted. This included inhaled fraction of oxygen (FiO_2_), positive end expiratory pressure (PEEP), minute volume (MV), and peak and mean airway pressures. In addition, the results of arterial blood gas (ABG) tests were logged. This was at three key moment—prior to the proning session, at the end of the proning session (i.e., before the patient was returned supine), and after the patient was placed back to the supine position.

The P/F ratio and A-a O_2_ gradient at each of the above points was calculated. Finally, the difference between various ABG values at each of these three key moments was examined. For each recorded value and index of oxygenation, the difference from the start to the end of the session of proning was calculated as was the difference between the moment prior to prone positioning and when the patient was replaced to the supine position.

The collected data were split into two sets. Most of the data (85%) were assigned to the training/development set. These data were used for hyperparameter tuning, and to create a final model fit (see below). The minority of the data (15%) were assigned to the testing/validation set. These data were isolated from model training and was used to assess the efficacy of the resultant model.

### Machine learning

All data processing, statistical calculations, and machine learning was performed in the R programming environment (version 4.2.2). The outcome that was predicted was death at less than or equal to seven full days after the end of an initial session of prone positioning.

Variables were analysed for missingness. Any variable with > 5% of instances missing was excluded completely from the analysis. A full overview of variables and missingness can be seen in the Supplement. Of those variables with ≤ 5% of values missing, all were determined to be Missing Completely At Random (MCAR). They were, therefore, deemed suitable for imputation, which was achieved using the bagged tree method [25]. Data were also screened to identify pairs of variables with a Pearson correlation coefficient > 0.9. If this was the case, one of those variables was removed.

Hyperparameters were tuned for optimal performance using the training set. The training data were divided using k-fold cross-validation where *k* = 5. A tuning grid of candidate values was calculated to investigate various combinations of hyperparameters according to maximum entropy sampling with 500 points [26]. Optimal hyperparameters were selected based on the best performing combination as measured by the resultant area under the Receiver Operating Characteristic curve (AUROC).

Modelling was achieved using the C5.0 algorithm [27, 28] with adaptive boosting [29]. This was executed through the `parsnip` package for R [29]. The C5.0 algorithm creates a final decision tree by iteratively splitting samples into subsets at each node, dividing subsets according to the variable that maximises information gain. It then removes (or “prunes”) the lowest level splits that do not contribute significantly. The adaptive boosting algorithm creates multiple decision trees, where later iterations focus on samples misclassified by earlier ones. The importance of a variable in C5.0 is related to the number of times the variable is used in classifying training data samples across the boosted trees. A final model was fit to the overall training data using these optimal hyperparameters. Predictions were made on the test set using this model. Performance metrics that were used to assess the performance of the model were sensitivity, specificity, and area under the Receiver Operating Curve (AUROC). Insight into the decision-making process of the resultant model was explored by interrogating SHAP (SHapley Additive exPlanations) values, which allows the contribution of each feature to a specific prediction to be quantified [31].

A final step that completes model validation by testing on data collected completely external to the original study was not performed in this proof-of-concept work.

## Results

### Patient population

**Fig. 1:**
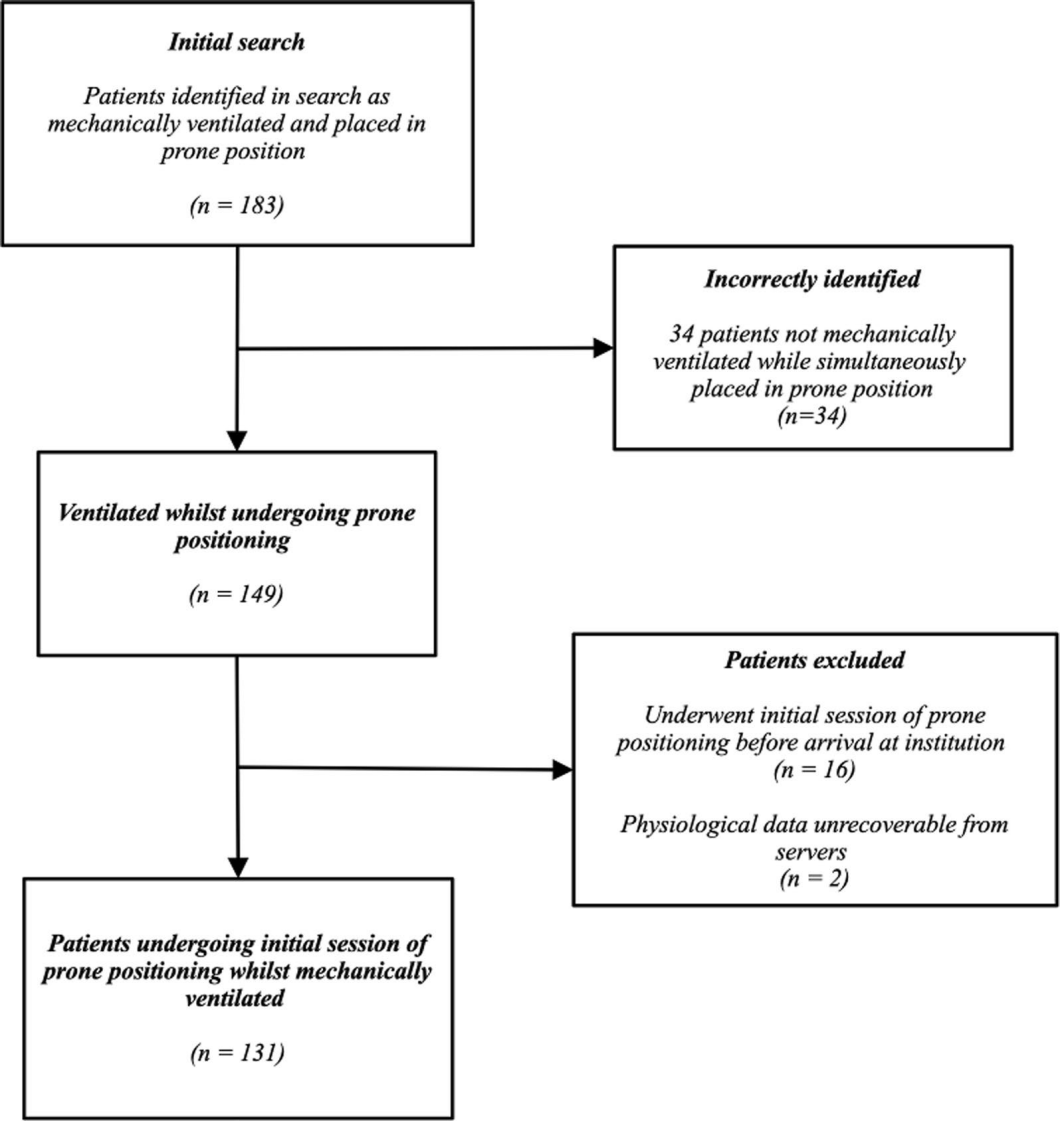
Selection of patient records for modelling process

A total of 183 records were identified during the search process. Of these, 54 were excluded, see Fig. 1 for overview. Of these, 131 cases were identified involving an initial session of prone positioning whilst intubated and mechanically ventilated. These patients were admitted to the ICU between 14/07/2013 and 20/09/2022. Patients were proned between 1 and 13 times with a median of 2 proning episodes. Most patients (*n* = 88) were male. The mean age of the sample population was 58.0 years (SD 15 years), and the mean APACHE II was 19.2 (SD 7.9). Most patients had “classic” ARDS (59%), with most others (38%) having ARDS secondary to COVID-19 (C-ARDS). Thirty-five patients (27%) died within 7 days of their initial session of prone positioning. An overview can be seen in Table 1.

**Table 1:** Demographic characteristics of patients undergoing first prone positioning

Characteristic	Measure (*n* = 131)
Gender	
Male	86 (66%)^1^
Female	44 (34%)^1^
Age (years)	58.0 (15.0)^2^
BMI	31.1 (7.6)^2^
Not recorded	24 (18%)^1^
ARDS source	
ARDS	81 (62%)^1^
C-ARDS	50 (38%)^1^
ARDS classification	
Mild	3 (2%)^1^
Moderate	54 (41%)^1^
Severe	74 (56%)^1^
Admitting location	
Ward	60 (46%)^1^
Emergency department (ED)	28 (21%)^1^
Inter-hospital transfer	36 (27%)^1^
Post-surgery	7 (5%)^1^
Length of stay in ICU (days)	13.3 (18.0)^3^
APACHE II	19.0 (7.9)^3^
Duration of initial proning (hours)	16.1 (3.6)^3^
Outcome	
Survived	72 (54%)^1^
Died	61 (46%)^1^
Died < 7 days of initial proning	35 (27%)^1^

^1^*n* (%)

^2^Mean (SD)

^3^Median (SD)

A comparison of demographic and physiological parameters at admission showed that the 7-day survivor and non-survivor groups are reasonably comparable but there were significant differences (Table 2). In particular, APACHE II scores were significantly different at admission (17.8 vs 22.8, *p* = 0.001), as were haemoglobin (12.4 g/dL vs 11.2 g/dL, *p* < 0.05), haematocrit (0.4 vs 0.3, *p* < 0.05), platelet count (246.3 × 10^9^/L vs 179.1 × 10^9^/L), and serum albumin (32.7 g/L vs 29.0 g/L, *p* < 0.01). Of relevance, indices of oxygenation were not significantly different at time of proning (Table 2).

**Table 2:** Key patient parameters immediately prior to initial proning session

	Death within 7 days of initial proning		
Parameter	No (*n* = 96)^1,2^	Yes (*n* = 35)^1, 2^	*p* value^3^
Demographics			
Age (years)	57.3 (54.2, 60.4)	60.9 (56.4, 65.4)	0.20
Weight (kg)	88.6 (84.6, 92.6)	82.2 (74.8, 89.6)	0.11
BMI (kg/m^2^)	30.8 (29.2, 32.4)*	32.3 (28.8, 35.8)*	0.40
APACHE II	17.8 (16.3, 19.3)	22.8 (19.9, 25.7)	0.001
Cardiovascular			
Systolic BP (mmHg)	123 (118, 129)	115 (107, 123)	0.10
MAP (mmHg)	82.1 (79.1, 85.1)	76.9 (73.4, 80.3)	0.06
HR (bpm)	92.0 (87.6, 96.5)	93.7 (85.8, 101.6)	0.70
Haematological			
Haemoglobin (g/dL)	11.6 (11.1, 12.1)	10.6 (9.8, 11.4)	0.036
Haematocrit	0.34 (0.31, 0.4)	0.32 (0.30, 0.35)	0.10
WCC (× 10^9^/L)	11.7 (10.5, 12.8)	14.1 (10.3, 17.9)	0.10
CRP (mg/L)	145 (121, 167)	157 (117, 197)	0.60
Respiratory			
FiO_2_	0.8 (0.70, 0.80)	0.80 (0.70, 0.80)	0.30
Resp. Rate (bpm)	20.7 (19.6, 21.8)	22.1 (20.5, 23.8)	0.20
Minute volume (L/min)	4.2 (3.3, 5.2)	3.2 (1.9, 4.4)	0.20
Paw (cmH_2_O)	7.0 (5.4, 8.6)	5.5 (2.9, 8.0)	0.30
Arterial blood gas (ABG)			
pH	7.34 (7.32, 7.36)	7.30 (7.26, 7.33)	0.039
PaO_2_ (kPa)	10.0 (9.5, 10.4)	9.1 (8.7, 9.6)	0.040
PaCO_2_ (kPa)	6.9 (6.6, 7.2)	7.7 (7.0, 8.3)	0.023
Bicarbonate (mmol/L)	28.1 (27.1, 29.2)	27.6 (25.3, 29.9)	0.60
Base Excess (mmol/L)	1.87 (0.72, 3.03)	0.94 (− 1.47, 3.35)	0.40
Sodium (mmol/L)	141 (140, 142)	141 (139, 142)	> 0.9
Potassium (mmol/L)	4.1 (4.0, 4.2)	4.3 (4.1, 4.5)	0.091
Indices of oxygenation			
P/F ratio (kPa)	14.0 (12.9, 15.2)	12.3 (11.0, 13.6)	0.083
A-a O_2_ gradient (kPa)	52.7 (49.4, 55.9)	55.9 (50.3, 61.5)	0.30

^*^ > 20% height values missing, BMI calculated with remainder

^1^*n* (%)

^2^Mean (SD)

^3^Two sample* t* test

### Machine learning

131 cases were included. The training set consisted of 110 cases, whilst the holdout set for final testing consisted of 21 cases. Three hyperparameters were tuned. These were number of trees, minimal node size, and proportion of observed samples used. Maximal model performance on the training set was found with number of trees equalling 7, a minimal node size of 11, and the use of 73.2% of observed samples. A figure showing model performance throughout the range of values for hyperparameter tuning on the training set can be seen in the Supplement.

**Fig. 2:**
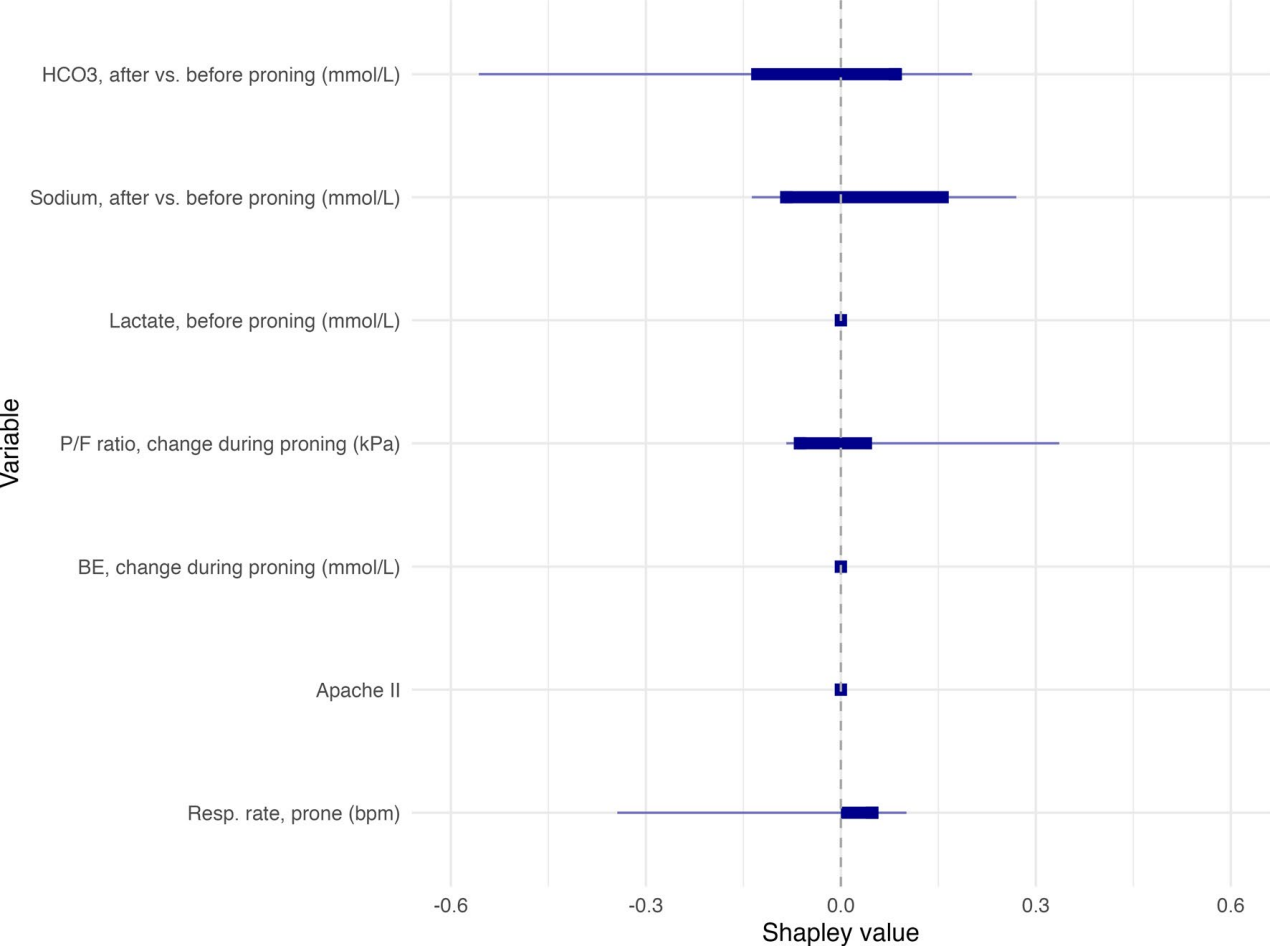
Aggregate SHAP values showing distribution of the contribution of each variable to predictions made by the model. The thicker line shows the interquartile range, while the thinner extends from the minimum to maximum values

The model isolated just seven variables from a total of 121 candidate parameters to make its predictions. The relative importance of these seven variables is shown in Table 3. A description of the individual decision trees that formed the final decision to be combined via boosting can be seen in the Supplement. The relative contribution of each of these variables can also be seen in Fig. 2, which visualises their relative contribution to the model’s predictions by computing aggregate SHAP values (Shapley Additive Explanations) on the testing set. The most significant impact was the patient’s respiratory rate when prone, APACHE II score, but the change in serum HCO3- and the change in P/F ratio before vs after proning was also important. The other three variables had relatively lower contribution to the model’s decision-making.

**Table 3:** Variable importance in final model

Variable	Relative importance (%)
Respiratory rate when prone (bpm)	100
Change in P/F ratio (kPa)	100
Change in base excess (mmol/L)	100
Apache II at admission	100
Lactate before proning (mmol/L)	76.2
Change in serum sodium (mmol/L)	68.8
Retained bicarbonate change (mmol/L)	68.2

**Fig. 3:**
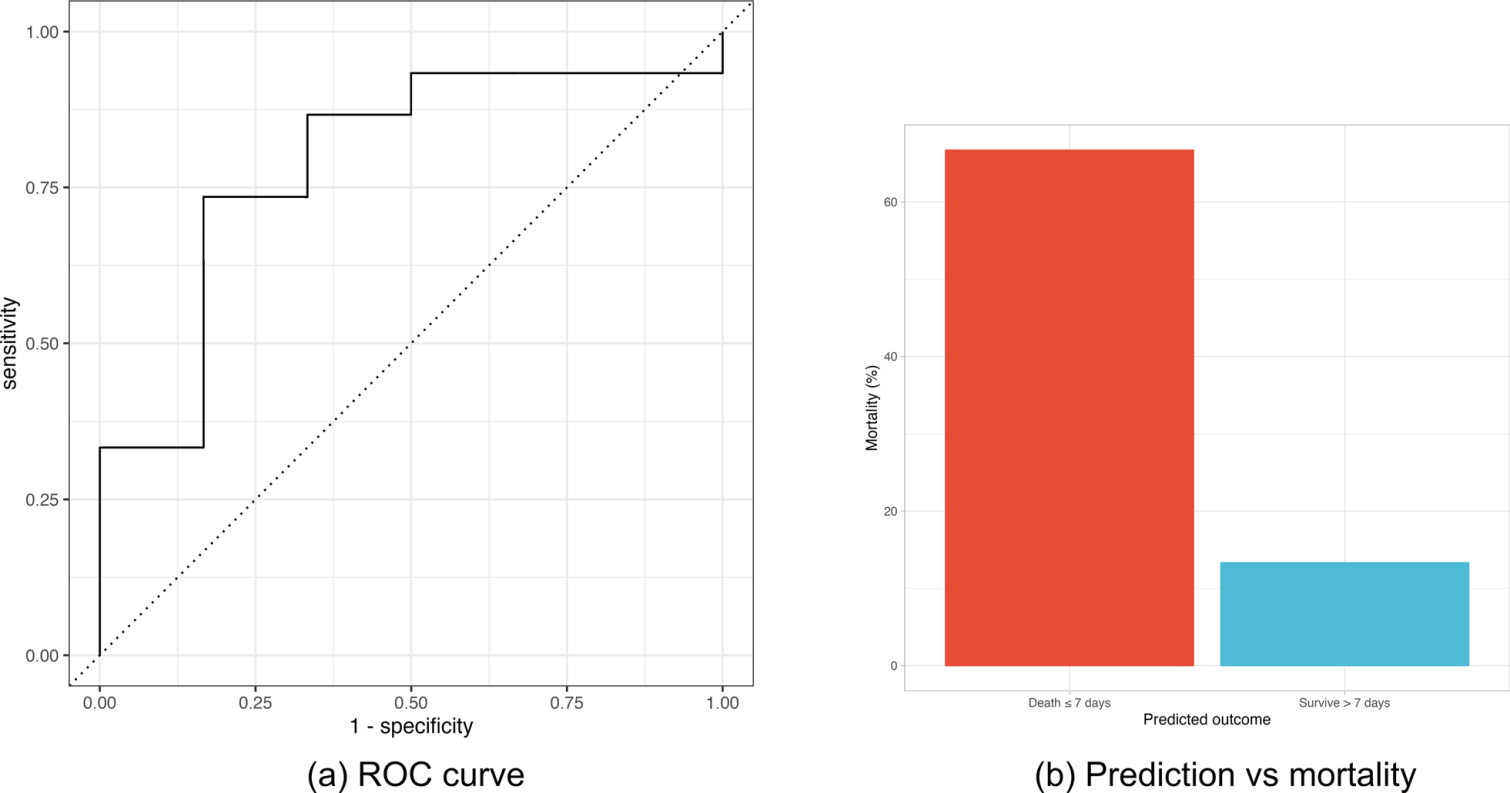
Panel showing combined figures of (**a**) ROC curve for final optimised model used to make predictions on the testing data and (**b**) predicted vs actual mortality for outcomes of model on test data

On the training data, the model produced an area under the receiver operating characteristic curve (AUROC) of 0.89 with a sensitivity of 0.83 and a specificity of 0.76. On the final testing data, the results showed an AUROC of 0.78 with a sensitivity of 0.80 and a specificity of 0.67 (Fig. 3a). An overview of these results is shown in Table 4.

**Table 4:** Performance metrics of C5.0 model

Metric	Training data	Test data
Sensitivity	0.83	0.80
Specificity	0.76	0.67
AUROC	0.89	0.78

A confusion matrix comparing model predictions to true outcome on the test cohort is visible in Table 5. In the test cohort, the overall 7-day mortality was 28.6%. In patients in whom the model predicted patient would survive to day 7 based on their response to proning, the mortality rate at day 7 was 13.3%. Conversely, if the model predicted the patient would not survive to day 7, their 7-day mortality rate was 66.67% (Fig. 3b). Error rates were 19.1% on the training data and 23.8% on the testing data.

**Table 5:** Confusion matrix showing true classification of patient outcome vs outcome predicted by model

		True outcome		
		Survived > 7 days	Died ≤ 7 days	Total
Predicted outcome	Survived > 7 days	12	2	14
	Died ≤ 7 days	3	4	7
	Total	15	6	21

**Fig. 4:**
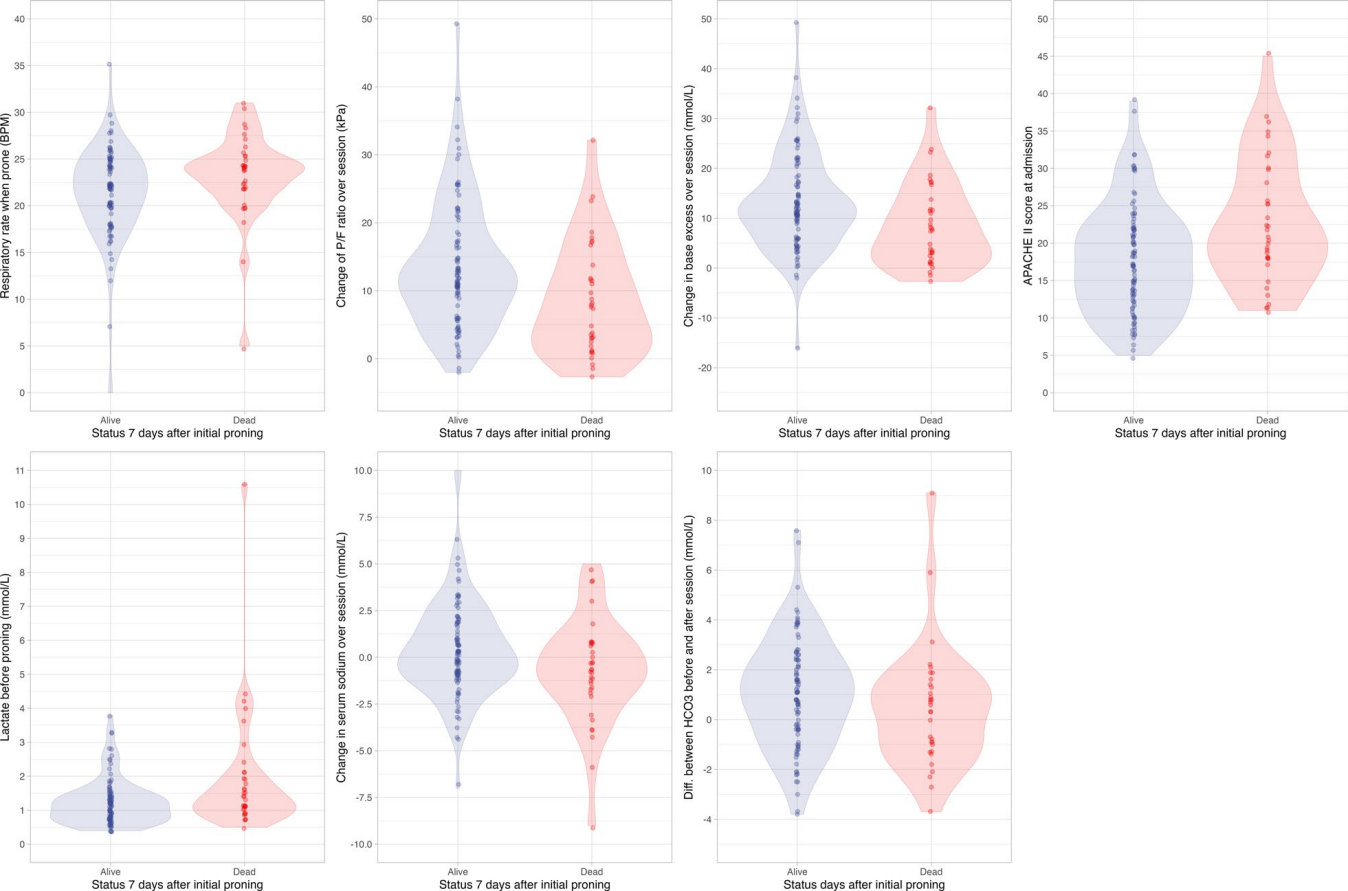
Violin plots comparing values for parameters identified as predictive by the boosted C5.0 model between patients who did and did not survive until 7 days following their initial session of prone positioning

The density distributions for the predictive variables, in both the training and test data, can be seen in the Supplement. These show similarity between the distribution of values between the training and test sets. A series of violin plots that show similarities between the distribution of predictive variables between patients who did and did not live beyond 7 days of their initial session of prone positioning can be seen in Fig. 4. Clear threshold values in any parameter that differentiate between patients who lived or died within 7 days of initial proning is not appreciable.

## Discussion

In this single-centre study we trained a C5.0 machine learning model with adaptive boosting to identify patients at high risk of death within 7 days based on their response to an initial management regimen that included prone positioning. In order to reach a decision, the model evaluated measures of the patient’s physiological status before undergoing the manoeuvre, and measures derived from serial ABGs taken before, during, and after proning. The model shows good overall performance (AUROC 0.78) with a high sensitivity and moderate specificity (0.80 and 0.67 respectively). The prediction model was developed using a limited data set (*n* = 131), and used commonly recorded information relating to the physiological, haematological, and biochemical parameters routinely collected in an ICU.

The model identified a cohort of patients with a higher mortality risk based on their general physiological status including their response to prone positioning. In the final test data, the group of patients that the model identified as surviving the 7 days following proning had a mortality of 13.3% while the group predicted to die within 7 days had a mortality of 66.7%, i.e., a fivefold higher mortality. We can consider these people ‘responders’ and ‘non-responders’ to prone positioning. As such, this approach could facilitate early identification of patients who may benefit from a detailed and thorough review of all aspects of their management to ensure complete optimisation. In addition, a consideration of other rescue therapies such as ECMO might be prompted [32]. For example, it is likely that early initiation of ECMO is most beneficial [16], and an accurate ability to identify these patients could prompt an evaluation of their suitability. Machine learning approaches have been used successfully to predict the development of ARDS [33], to examine the connection between mechanical power and mortality [34], and to identify phenotypes within ARDS that have different response to treatment [20]. This shows a clear potential role for these techniques to prognosticate and guide management. To our knowledge, the work presented here is the first to predict 7-day mortality in this context.

The model isolated only seven variables to make final predictions from 121 candidate variables. Some of these have evidence that supports their importance in relation to outcome. However, it must be stated strongly and definitively that it is not possible to draw any conclusions about causality from this approach. The model’s use of certain parameters and thresholds does not imply that there is a causal link between these factors or their bearing on mortality, nor that the modification of these variables will result in a different outcome. The techniques used in this process are based in statistical, rather than clinical, reasoning [35]. Given the importance of interpretability, modelling can also be approached from the point of view of a mathematical description of physiology [36]. Such approaches can allow results to be understood in much more explicit and pathophysiologically interpretable ways [37].

The C5.0 modelling approach produces a decision tree structure [27], but the use of adaptive boosting complicates simple interpretation, which produces an ensemble of decision trees that are all given a weighted vote towards a final classification [38]. In the case of a C5.0 model, variable importance (Table 3) refers to the percentage of training set samples that fall into all the terminal nodes after the split. SHAP values also provide a powerful tool for interpreting machine learning models, as they quantify how much each of the input variables contribute to the predictions made by the model. Such insights into interpretability (or lack thereof) can raise important ethical and legal considerations regardless of a clinician’s willingness to trust such a tool [39]. Although SHAP values aid model interpretability, they do not give any insight into causality and must not be interpreted as such [40].

Our model demonstrates that the Apache II score is the most influential predictor of short-term mortality among patients in the prone position. The respiratory rate of the patient whilst in the prone position was also significant. A small change in serum bicarbonate when the patient was returned supine is also a significant contributor to the model's predictions. A significant contribution is also due to the improvement in P/F ratio throughout the proning session. Serum sodium, lactate, and base excess (BE) are minor contributors. With regards to the P/F ratio, in patients with ARDS secondary to COVID-19, an initial improvement in P/F ratio has been shown to be prognostic of increased survival [41]. However, this is not always the case with ARDS due to other causes, when changes in P/F ratio in response to the first session of prone positioning had poor prognostic value [42, 43]. Likewise, the presence of the APACHE II score is plausible given its proven association with ICU mortality [44, 45]. Both hyper and hyponatraemia are associated with increased mortality in ICU patients, and changes to serum sodium over a 48-h period are also associated with increased mortality [46]. In the sample of patients in this study, the mean change of sodium over the course of proning was + 0.14 mmol/L. However, two of the patients who died within 7 days of initial proning had a decrease > 5 mmol/L. Such datapoints can assume importance in predictive models. It is interesting to note that some variables (for example, change in serum sodium) had close to zero direct contribution to model decision as measured using SHAP values, but nonetheless had higher variable importance scores. Such variables may have indirect and complex connections to the outcome that are mediated through other variables in the model. The nature of how these elements contribute to a final classification decision by the model can be seen in the Supplement.

The absence of some factors that have been shown to be prognostic in this context, despite their presence in the data, is also of note. For example, it has been previously reported that a decrease in PaCO_2_ during proning has prognostic value for a positive outcome [47], but this variable was not deemed to be necessary to predict outcome by the model in this study.

While the APACHE II score is a well-established tool for predicting mortality in critically ill patients, our C5.0 model offers several advantages. Despite a statistically significant difference in mean APACHE II scores between survivors and non-survivors (17.8 vs 22.8, *p* = 0.001), the distributions of these scores overlapped considerably (Fig. 4). This indicates that the APACHE II score alone may not be sufficient to accurately predict mortality in these specific cases. Our C5.0 model, by incorporating a broader range of patient characteristics and leveraging machine learning techniques, demonstrates superior predictive performance compared to the APACHE II score.

To assess the performance of our proposed C5.0 model with adaptive boosting, we also trained several alternative machine learning models. Logistic regression with Elastic Net regularization and a Gaussian Naive Bayes model were both considered. While these models demonstrated reasonable performance, particularly in terms of sensitivity, their overall predictive capabilities were inferior to our C5.0 approach. The logistic regression model, despite tuning its hyperparameters, exhibited a relatively low AUROC of 0.58. The Gaussian Naive Bayes model, while achieving a higher AUROC of 0.73, still fell short of the performance metrics attained by our C5.0 model, suggesting that the C5.0's ability to handle non-linear relationships is important for accurately predicting short-term mortality in this patient population. Other studies have also reported a superior performance of the C5.0 algorithm when compared with logistic regression models in a similar context [48]. Attempts were also made with XGBoost, Random Forest, and Support Vector Machines. These models generally showed perfect sensitivity and very poor specificity. More details can be seen in the Supplement.

There are several important limitations to this study. First, this is a retrospective analysis of prospectively collected clinical data, which can be problematic when used to construct machine learning models for predicting outcomes. Retrospective data sets are susceptible to various biases inherent in historical data collection practices, including selection bias and information bias [48]. These biases can distort the model’s “understanding” of relationships between variables. Other problems relate to inconsistencies and inaccuracies in medical records due to differences in documentation and recording practices over time. Consequently, models built on such data might perform well in historical testing but fail to generalize to contemporary patient populations, undermining their clinical utility and reliability in predicting future outcomes.

Currently, using the model requires a cumbersome process of isolating variables, creating a new R dataframe, and running the fitted model. This 11-variable approach is impractical for busy ICUs. Integrating the model directly into a clinical information system (CIS) would be more feasible but requires significant logistical effort to validate and implement.

Using a small data set to train a machine learning model poses several significant challenges. Small data sets are prone to overfitting, where the model learns noise and specific patterns in the training data rather than generalisable trends [49]. This can result in a model that performs well on the provided data but fails to predict accurately on new, unseen data. Small data sets are more susceptible to random fluctuations and outliers, which can disproportionately influence the model's parameters and skew predictions. Moreover, a limited sample size might not capture the full spectrum of patient variability and clinical scenarios, potentially limiting the model's ability to accurately predict outcomes in more diverse populations. To mitigate these concerns, future studies should aim to collect larger data sets to improve the model's generalisability and enhance its clinical applicability. However, the C5.0 algorithm has been shown to perform well in small data sets comparable in size to this study [50]. Despite these issues, our model performed reasonably. The C5.0 model achieved error rates of 19.1 and 23.8% on the training and test data sets, respectively. While these rates are relatively low, they do indicate a degree of overfitting, as the model's performance is slightly better on the training data. This is a common phenomenon in machine learning and can be addressed through techniques like cross-validation or regularization. However, given the complexity of predicting short-term mortality in critically ill patients, an error rate of around 20% can be considered reasonable. Similar error rates have been reported in previous studies using machine learning models for predicting mortality in intensive care units [51].

Data that are missing or absent are an issue. The servers at the institution in which the study was performed do not yet permanently record all bedside data. A full overview of all the parameters that were recorded and collected for the study can be seen in the Supplement. While several additional variables, such as plateau pressure and mechanical power, could have potentially enhanced the model's predictive capabilities, their inclusion was hindered by the limitations of the ICU's data collection system. Future studies with more comprehensive data collection could explore the potential benefits of incorporating these variables into the model.

The TRIPOD statement emphasises the importance of using external data to fully validate a predictive model [21]. We present this work as an initial proof-of-concept. The primary objective of this phase was to demonstrate the feasibility of using machine learning to predict medical outcomes within the constraints of available data. Even with these limitations, the model has shown promising performance, indicating potential for further development. To ensure effective validation and implementation, our future work will focus on applying the model to external data sets from similar clinical environments. Specifically, our aim is to validate the model in ICUs with comparable patient demographics, disease profiles, and care practices. This will be performed using external data sets that are openly available as the next step in model development, for example the MIMIC-IV, eICU-CRD, and SICdb [52–54].

## Conclusion

Our study demonstrates the potential of a boosted C5.0 algorithm to predict short-term mortality in patients undergoing an initial management regimen that includes prone positioning, by integrating a constellation of variables relating to underlying physiology and response to proning. In doing so, the model identifies a cohort who are at higher risk of early death. This work shows the potential that the use of these models at the bedside could identify patients early in their clinical course at high mortality risk and prompt a detailed and thorough review of their management. This proof of concept represents an important first step in leveraging machine learning in identifying patients with ARDS at high risk for early mortality.

## Supplementary Information

Additional file 1.

## Data Availability

The data sets used and/or analysed during the current study are available from the corresponding author on reasonable request.
