# Supplementary material for: The development of a C5.0 machine learning model in a limited data set to predict early mortality in patients with ARDS undergoing an initial session of prone positioning
Source: Intensive Care Med Exp. 2024 Nov 14;12:103. doi: 10.1186/s40635-024-00682-z (PMC11564488; doi:10.1186/s40635-024-00682-z)
Supplement: Supplementary file 1 — Additional file 1. [file 40635_2024_682_MOESM1_ESM.docx]

**SUPPLEMENTAL MATERIAL**

**Title:** The development of a C5.0 machine learning model in a limited dataset to predict early mortality in patients with ARDS undergoing an initial session of prone positioning.

**Authors:** David M. Hannon^1,2^, Jaffar David Abbas Syed^1^, Bairbre McNicholas^1,2^, Michael Madden^3^, John G. Laffey^1,2^

**Author Affiliations**

1: Department of Anaesthesia, Galway University Hospital, and School of Medicine, University of Galway, Ireland.

2: Anaesthesia and Intensive Care Medicine, School of Medicine, University of Galway, Galway, Ireland.

3: School of Computer Science, University of Galway, Galway, Ireland.

**Corresponding author:** David M. Hannon BSc MB BCh BAO FCAI, Anaesthesia and Intensive Care Medicine, School of Medicine, University of Galway, Galway, Ireland. E-mail: d.hannon8@universityofgalway.ie

**Analysis of A-a O_2_ gradient throughout first prone positioning**

An examination of A-a O_2_ gradient showed that comparison of means between patients who did and did not die within 7 days of their initial session of prone positioning showed that patients who died within 7 days of prone position showed a smaller improvement in A-a O_2_ gradient from baseline (-19.9kPa vs -29.1, p=0.007) as well as a higher value at the end of proning (36.1kPa vs 23.9kPa, p=<0.001) than patients who survived. Other comparisons did not reach statistical significance. These results can be seen in **Table S1** and in **Figure S1**.

**Table S1**: Comparison of A-a O_2_ gradient (kPa) values throughout initial prone positioning.

| **Patient position** | **A-a gradient (kPa) -**  **Died ≤ 7 days^1, 2^** | **A-a gradient (kPa) -**  **Lived > 7 days^1, 2^** | ***p*-value^3^** |
| --- | --- | --- | --- |
| Before proning (supine) | 55.9 (50.3, 61.5) | 52.7 (49.4, 55.9) | 0.3 |
| At end of proning (prone) | 36.1 (28.8, 43.4) | 23.9 (21.5, 26.3) | <0.001 |
| After proning (supine) | 42.9 (35.8, 49.9) | 37.3 (33.9, 40.8) | 0.12 |
| Change throughout proning | -19.9 (-26.2, -13.5) | -29.1 (-32.5, -25.7) | 0.007 |
| Final value relative to baseline | -13.1 (-19.5, -6.7) | -15.5 (-19.4, -11.6) | 0.5 |
| ^1^ Mean |  |  |  |
| ^2^ CI = Confidence Interval (95%) |  |  |  |
| ^3^ Two Sample *t*-test |  |  |  |

**Figure S1**: Graphs displaying the change in absolute values of A-a O2 gradient through initial proning.


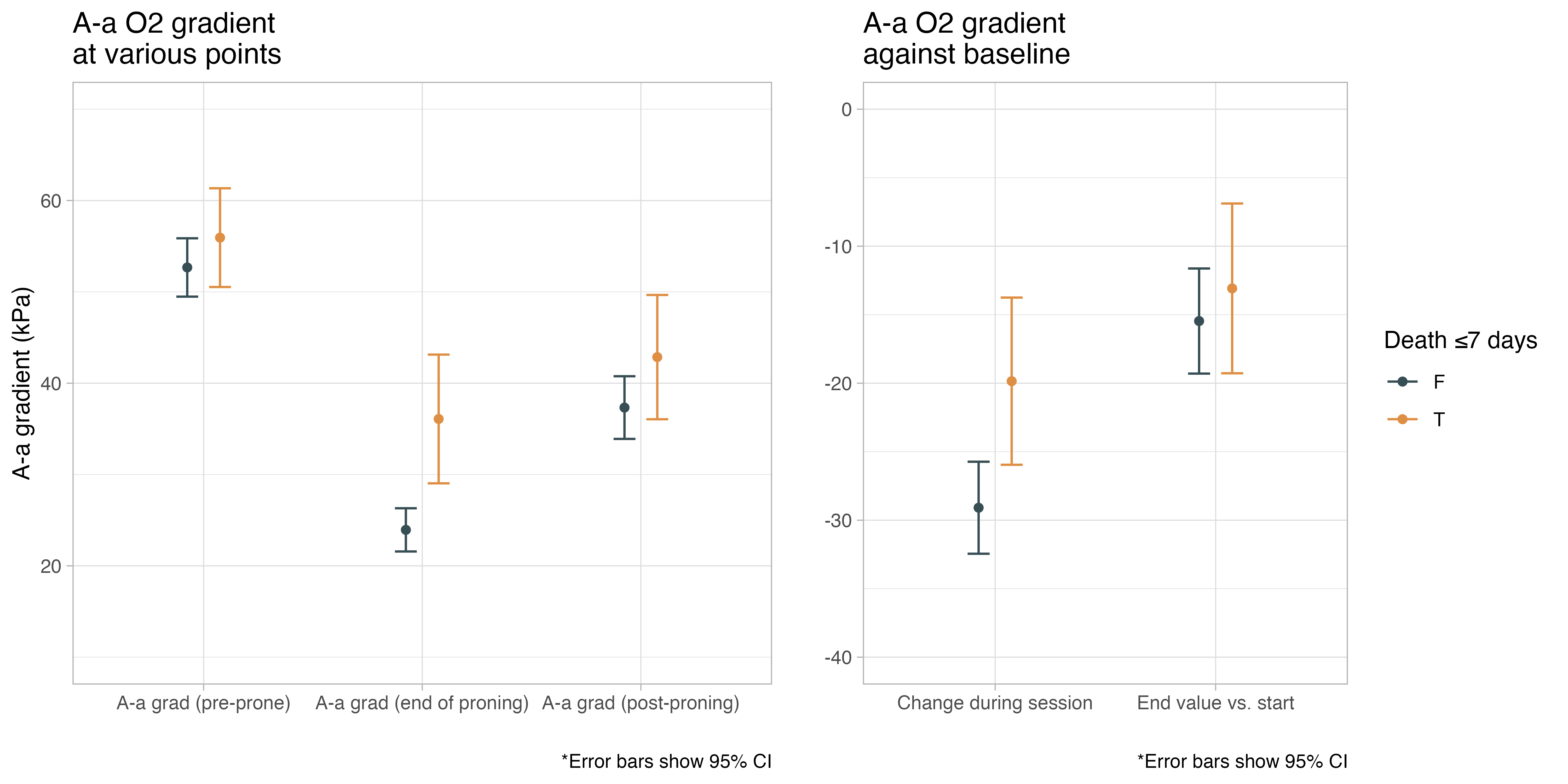


**Analysis of P/F ratio throughout first prone positioning**

**Table S2**: Comparison of P/F ratio (kPa) values throughout initial prone positioning.

| **Patient position** | **PFR (kPa) -**  **Died ≤ 7 days^1, 2^** | **PFR (kPa) -**  **Lived > 7 days^1, 2^** | ***p*-value^3^** |
| --- | --- | --- | --- |
| Before proning (supine) | 12.3 (11.0, 13.6) | 14.0 (12.9, 15.1) | 0.08 |
| At end of proning (prone) | 20.9 (17.4, 24.4) | 27.2 (25.3, 29.1) | 0.001 |
| After proning (supine) | 16.6 (13.7, 19.4) | 19.4 (17.3, 21.4) | 0.14 |
| Change throughout proning | 8.6 (5.8, 11.4) | 13.2 (11.2, 15.2) | 0.01 |
| Final value relative to baseline | 4.3 (2.0, 6.6) | 5.3 (3.4, 7.2) | 0.5 |
| ^1^ Mean |  |  |  |
| ^2^ CI = Confidence Interval (95%) |  |  |  |
| ^3^ Two Sample *t*-test |  |  |  |


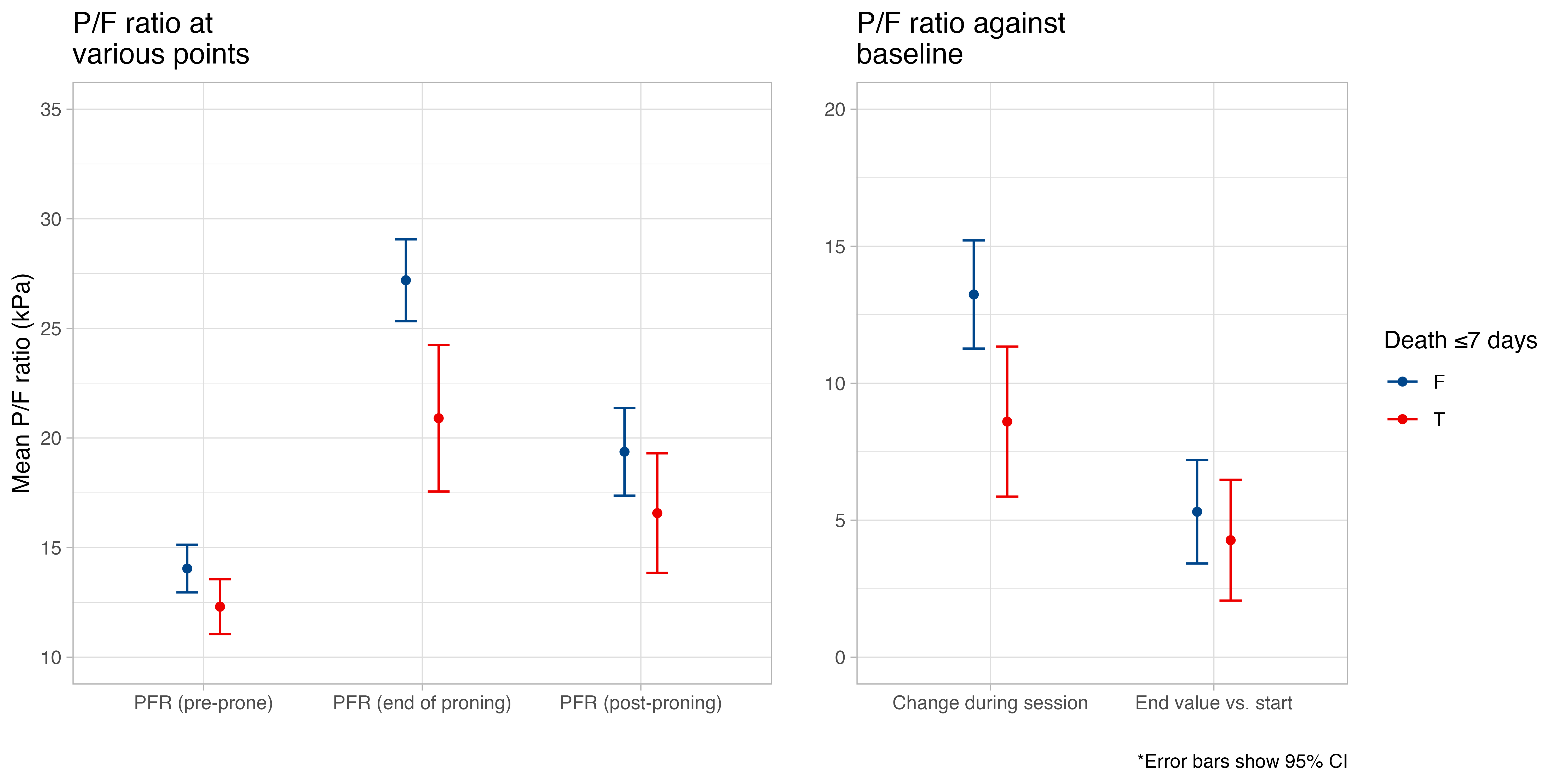
**Figure S2:** Graphs displaying the change in values of P/F ratio through initial proning.

**Analysis of PaCO_2_ throughout first prone positioning**

These results can be seen in **Table S2** and in **Figure S2**.

**Table S3**: Comparison of PaCO_2_ (kPa) values throughout prone positioning.

| **Patient position** | **PaCO_2_ (kPa) -**  **Died ≤ 7 days^1, 2^** | **PaCO_2_ (kPa) -**  **Lived > 7 days^1, 2^** | ***p*-value^3^** |
| --- | --- | --- | --- |
| Before proning (supine) | 7.7 (7.0, 8.4) | 6.9 (6.6, 7.2) | 0.023 |
| At end of proning (prone) | 7.0 (6.5, 7.5) | 6.5 (6.2, 6.8) | 0.2 |
| After proning (supine) | 7.1 (6.6, 7.6) | 6.6 (6.4, 6.9) | 0.066 |
| Change throughout proning | -0.7 (-1.2, -0.2) | -0.4 (-0.7, -0.1) | 0.3 |
| Final value relative to baseline | -0.6 (-1.0, -0.1) | -0.3 (-0.5, 0.0) | 0.3 |
| ^1^ Mean |  |  |  |
| ^2^ CI = Confidence Interval (95%) |  |  |  |
| ^3^ Two Sample *t*-test |  |  |  |

**Figure S3:** Graphs displaying the change in absolute values of PaCO_2_ gradient through initial proning.


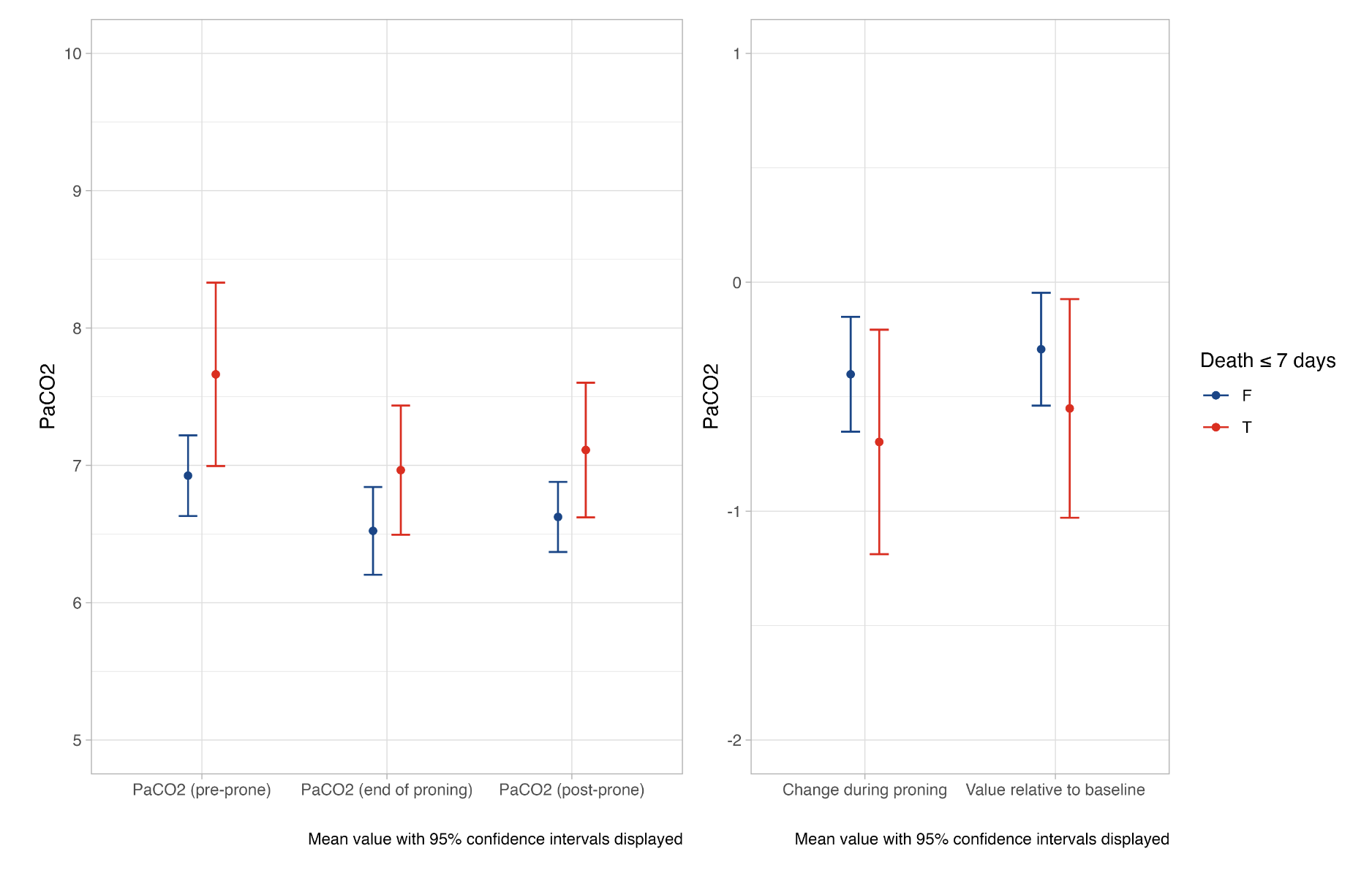


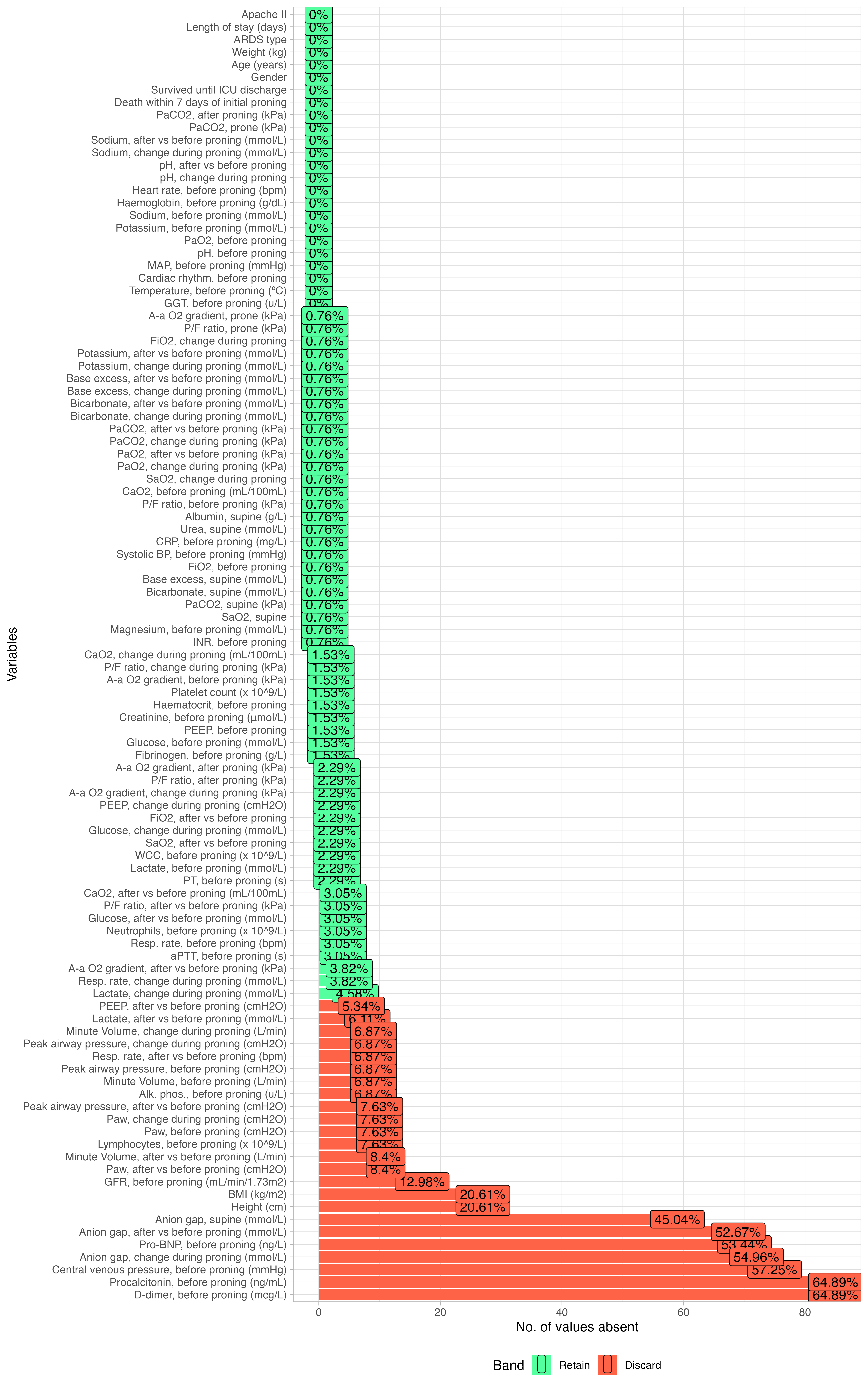


**Figure S4:** Full list of captures and calculated variables evaluated in model. Variables with >5% datapoints missing were excluded, otherwise BTI was used

| **Characteristic** | **Survived ≥ 7 days after proning^1, 2^** | **Died <7 days after proning^1, 2^** | **p-value^3^** |
| --- | --- | --- | --- |
| Age (years) | 57.3 (15.3) | 60.9 (13.0) | 0.2 |
| BMI | 30.8 (7.0) | 32.3 (9.1) | 0.4 |
| Gender | M = 68%  F = 32% | M = (50%)  F = (50%) | 0.3  - |
| Apache II | 17.8 (7.3) | 22.8 (8.4) | 0.001 |
| Heart rate (bpm) | 94.6 (24.4) | 97.8 (22.0) | 0.5 |
| MAP (mmHg) | 87.0 (17.1) | 92.7 (25.9) | 0.15 |
| Inotropes in progress | 42 (44%) | 13 (37%) | 0.6 |
| FiO_2_ | 0.7 (0.2) | 0.7 (0.2) | 0.9 |
| P/F ratio (kPa) | 18.0 (9.7) | 17.1 (8.1) | 0.6 |
| A-a O_2_ gradient (kPa) | 47.4 (17.9) | 49.3 (19.4) | 0.6 |
| pH | 7.4 (0.1) | 7.4 (0.1) | 0.6 |
| PaO_2_ (kPa) | 11.9 (7.1) | 11.1 (4.0) | 0.5 |
| PaCO_2_ (kPa) | 5.8 (1.6) | 5.7 (1.8 | 0.8 |
| SaO_2_ (%) | 95.1 (3.0) | 94.7 (4.8) | 0.6 |
| Sodium (mmol/L) | 138.6 (5.1) | 138.4 (5.8) | 0.8 |
| Potassium (mmol/L) | 3.9 (0.7) | 3.8 (0.8) | 0.3 |
| Glucose (mmol/L) | 8.9 (3.4) | 8.3 (4.0) | 0.4 |
| Lactate (mmol/L) | 1.6 (1.4) | 1.6 (2.0) | >0.9 |
| Haemoglobin (g/dL) | 12.4 (2.6) | 11.2 (2.7) | 0.026 |
| Haematocrit | 0.4 (0.1) | 0.3 (0.1) | 0.014 |
| WCC (x 10^9^/L) | 12.1 (6.6) | 12.0 (8.6) | >0.9 |
| Platelets (x 10^9^/L) | 246.3 (131.7) | 179.1 (149.1) | 0.015 |
| CRP (mg/L) | 112.4 (104.1) | 136.3 (108.3) | 0.3 |
| Urea (μmol/L) | 9.1 (6.4) | 10.7 (8.3 | 0.2 |
| Creatinine (μmol/L) | 106.9 (87.5) | 116.6 (88.3) | 0.6 |
| Albumin (g/L) | 32.7 (6.4) | 29.0 (5.7) | 0.004 |

^1^ Mean (SD); n (%)

^2^ CI = Confidence Interval

^3^ Two Sample t-test; Pearson’s Chi-squared test

**Table S4**: Patient characteristics at ICU admission

**Predictive variable comparison**


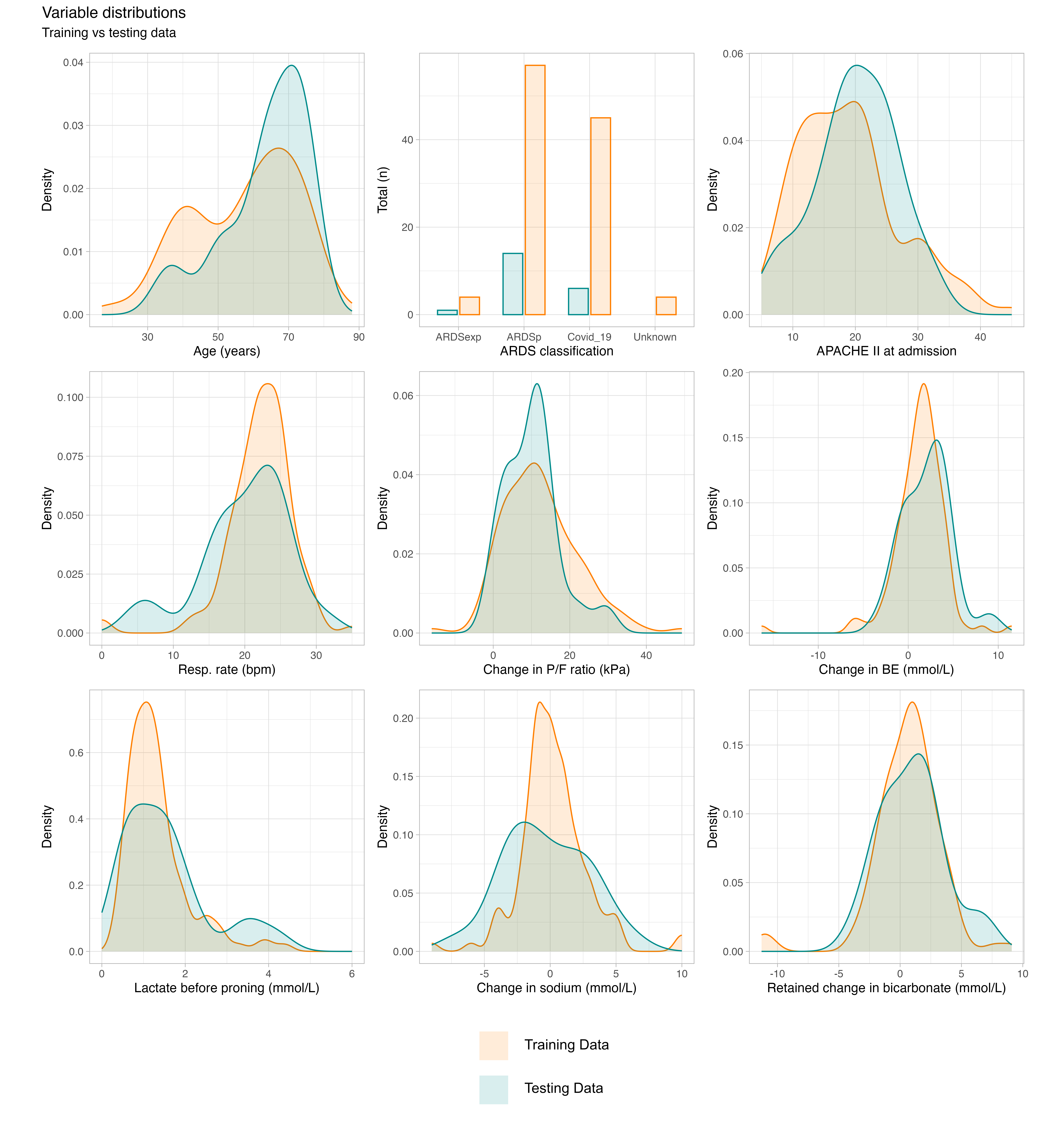
**Figure S5:** Comparison of density distribution for training (yellow) and testing (blue) data used in model training and validation process

**Decision trees within C5.0 model**

Class specified by attribute `outcome'. Read 80 cases (92 attributes) from undefined.data

----- Trial 0: -----

Decision tree:

pfr_change_absolute <= 3.802564: T (14/5)

pfr_change_absolute > 3.802564: F (66/10)

----- Trial 1: -----

Decision tree:

resp_rate_prone <= 21: F (24.9/0.8)

resp_rate_prone > 21:

:...bicarbonate_retain_absolute <= 2.2: T (43.4/18.6)

bicarbonate_retain_absolute > 2.2: F (11.7)

----- Trial 2: -----

Decision tree:

resp_rate_prone <= 19: F (13.3)

resp_rate_prone > 19:

:...apache_ii <= 21: F (45.3/8.6)

apache_ii > 21: T (21.4/8.2)

----- Trial 3: -----

Decision tree:

base_excess_change_absolute > 3.1: F (14.6)

base_excess_change_absolute <= 3.1:

:...lactate_abg_supine <= 0.8: F (17.6/2.6)

lactate_abg_supine > 0.8: T (47.8/17.6)

----- Trial 4: -----

Decision tree:

F (80/28.5)

----- Trial 5: -----

Decision tree:

apache_ii <= 14: F (15.9)

apache_ii > 14:

:...sodium_abg_change_absolute <= 1: T (49/19.4)

sodium_abg_change_absolute > 1: F (11)

**SHAP value breakdown for exemplar prediction**

The image below outlines the SHAP values leading to the model’s prediction for a single case. In this case, the true outcome was that the patient lived beyond 7 days following initial proning (ie the predicted outcome was FALSE). The predicted likelihood of 0.248 fell below the classification threshold, so the model prediction was also FALSE.

**
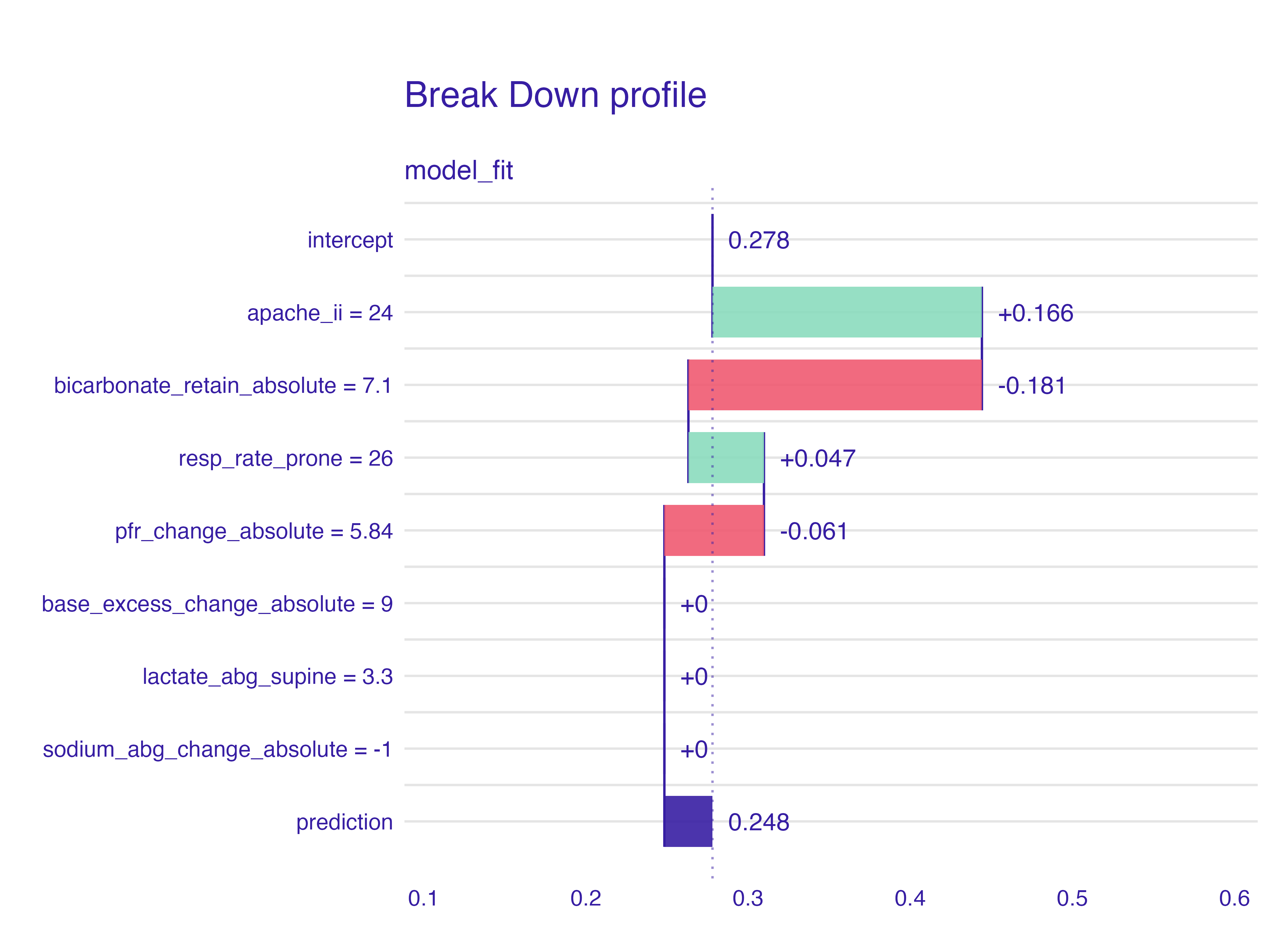
**

**Figure S6: E**xample prediction

**Results of hyperparameter tuning**


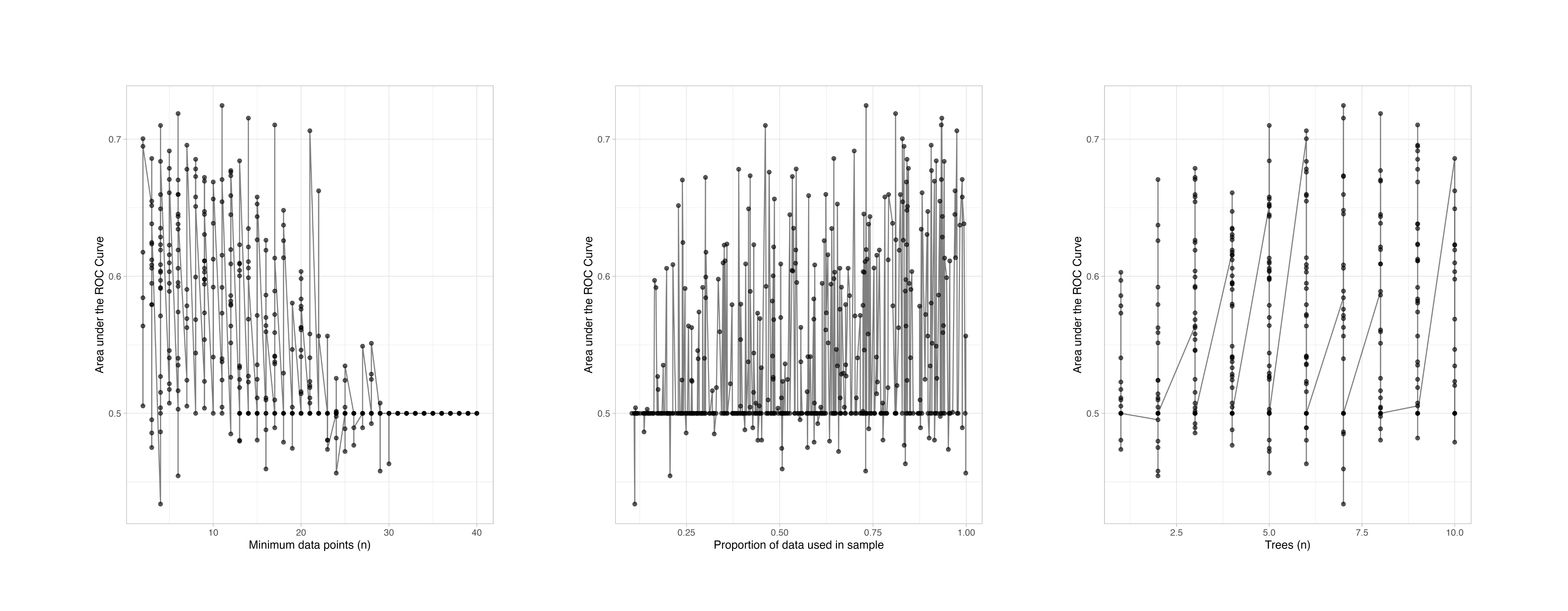
**Figure S7:** Results of hyperparameter tuning. Graphs show AUROC resulting from various hyperparameter values used on training data.

**Metrics for other ML models attempted**

|  | **Training data** | | |  | **Testing data** | | |
| --- | --- | --- | --- | --- | --- | --- | --- |
| **Model** | **Sensitivity** | **Specificity** | **AUROC** |  | **Sensitivity** | **Specificity** | **AUROC** |
| GNB | 0.82 | 0.51 | 0.73 |  | - | - | - |
| LR | 1.0 | 1.0 | 1.0 |  | 0.8 | 0.5 | 0.58 |
| XGBoost | 1.0 | 0.10 | 0.81 |  | 1.0 | 0.0 | 0.74 |
| SVM | 1.0 | 0.12 | 0.83 |  | 1.0 | 0.0 | 0.74 |
| RF | 1.0 | 1.0 | 1.0 |  | 1.0 | 0.17 | 0.76 |
